# Geriatric Approaches to Rectal Cancer: Moving Towards a Patient-Tailored Treatment Era

**DOI:** 10.3390/jcm14041159

**Published:** 2025-02-11

**Authors:** Carlo Vallicelli, Silvia Jasmine Barbara, Elisa Fabbri, Daniele Perrina, Giulia Griggio, Vanni Agnoletti, Fausto Catena

**Affiliations:** 1 General, Emergency and Trauma Surgery Department, Maurizio Bufalini Hospital, Viale Ghirotti 286, 47521 Cesena, Italy; daniele.perrina@auslromagna.it (D.P.); giulia.griggio@outlook.it (G.G.); fausto.catena@auslromagna.it (F.C.); 2 Department of Morphology, Experimental Medicine and Surgery, University of Ferrara, 44121 Ferrara, Italy; sissibarbara@gmail.com; 3 Department of Medical and Surgical Sciences, University of Bologna, 40126 Bologna, Italy; elisa.fabbri0909@gmail.com; 4 Anesthesiology and Intensive Care Unit, Maurizio Bufalini Hospital, 47521 Cesena, Italy; vanni.agnoletti@auslromagna.it

**Keywords:** rectal cancer, geriatric management of rectal cancer, geriatric surgery, radical and/or radiation therapy

## Abstract

Rectal cancer is a significant global health concern, particularly amongst the elderly population, with rectal cancer accounting for approximately one-third of cancer cases in this population. Older adults often present with advanced disease stages and unique clinical manifestations, such as tumors closer to the anal verge and with greater size. Diagnosis typically involves a series of screening and imaging strategies, culminating in accurate staging through pelvic MRI, endoscopic ultrasound, and CT scan. Management of rectal cancer in older adults emphasizes individualized treatment plans that consider both the cancer stage and the patient’s overall health status, including frailty and comorbidities. A multidisciplinary approach, including a mandatory geriatric assessment, is essential for optimizing outcomes, in order to improve survival and quality of life for elderly patients with rectal cancer.

## 1. Introduction

Colorectal neoplastic disease, which includes rectal cancer (that accounts for approximately one-third of colorectal malignancies),is the fourth most commonly diagnosed cancer worldwide, regardless of gender. The incidence of colorectal cancer is predicted to increase, with 2.2 million new cases expected per year by 2030, reflecting a further 20% surge. Despite a downward trend in mortality rates over recent decades, primarily attributed to enhanced screening campaigns and improved treatment options, rectal cancer remains one of the leading causes of cancer-related deaths. The median age at diagnosis is around 70 years of age, so this is a disease that predominantly affects older adults [1]. Risk factors for rectal cancer include tobacco and alcohol use, type II diabetes, high body mass index (BMI), inflammatory bowel disease (IBD), and high red meat consumption. Conversely, protective factors appear to be associated with the intake of non-steroidal anti-inflammatory drugs (NSAIDs), as well as a diet rich in fiber, fruits, milk, and garlic. Colon and rectal cancers share a variety of genomic mutations. However, rectal cancer predominantly develops through chromosomal instability or altered mismatch repair systems. Only a small percentage of cases have hereditary origins, such as Lynch syndrome and familial adenomatous polyposis syndrome.

Colon and rectal cancers share a variety of genomic mutations; however, rectal cancer predominantly develops through chromosomal instability or altered mismatch repair systems. Only a small percentage of cases have hereditary origins, such as Lynch syndrome and familial adenomatous polyposis syndrome

Dealing with older adults diagnosed with rectal cancer is often challenging. Chronological age alone is known to not be enough to comprehensively evaluate the patient and to enable proper decision-making. Moreover, these patients are often not included in the clinical trials that support current guidelines for “ideal” patients. Older adults are sometimes excluded from screening protocols at an age that varies according to the organization of the health system. It is no surprise, then, that with increasing age, there is a reported increase in rectal cancers that are closer to the anus and larger in size, making their management more complex [2]. Therefore, there is a clear need to tailor every single treatment to every single patient, and to consider frail older adults with rectal cancer as a different subset of patients as compared with the “ideal” ones undergoing the gold-standard treatment strategy.

## 2. Clinical Manifestations and Diagnosis

There are no specific diagnostic signs and symptoms for rectal cancer, but they can strongly suggest its presence. Older patients (aged over 65) may refer to rectal bleeding and/or changes in bowel habits lasting for more than 6 weeks. Due to physiological changes, these patients may have or may experience abdominal pain, mucorrhoea, and weight loss, albeit potentially without significant pain. The association of one or more clinical symptoms and signs must prompt healthcare providers to initiate a diagnostic workup [3].

It is recommended that patients between 50 and 69 undergo a fecal occult blood test every two years, while those aged 50 to 64 should undergo colonoscopy at least once in their lifetime [4]. Experts advise against conducting unselected screening in patients over 85, as the risk-to-benefit ratio, life expectancy, and cost-effectiveness become less favorable [5].

However, the interruption of screening protocols in Western countries happens at an average age, often much lower than 85, varying according to the health system’s organization. Moreover, in frail older adults, family support is frequently weak or lacking, making early diagnosis difficult.

It is no surprise, then, that there is a higher rate of massive rectal cancers closer to the anal verge in older adults.

A 12-year Australian prospective database of 699 rectal cancer patients reported data after patient stratification by three age groups: ≤65 years, 66–79 years, and ≥80 years. The number of patients aged 80 years or older was 118 (17%). The authors reported that with increasing age, rectal cancers were significantly closer to the anal verge. Moreover, for patients undergoing surgery, there was a significant association between age and an increase in rectal cancer size [2].

Despite the more complex situations clinicians face in frail older adults, diagnostic tools to diagnose and stage rectal cancer are the same as for “ideal” younger patients. However, the utilization of each tool in frail older adults should be accurately evaluated, according to the expected tolerance of each patient and the expected impact of this on the decision-making process.

Proctoscopy determines the dimension and length of the cancer and its distance from the anal verge. A total colonoscopy is valuable for detecting possible synchronous lesions in up to 30% of cases, or for identifying other pathological conditions [6]. Lesions are classified into three groups based on their distance from the anal margin (AM). Lesions within 5 cm from the AM are low rectal cancers, those extending from 5 to 10 cm are classified as middle rectal cancers, and those from 10 to 15 cm are considered high rectal cancers. Lesions found above 15 cm belong to colon cancer [7,8]. High-risk histological features include positive margins, lymphovascular invasion, poor tumor differentiation, and submucosal invasion. All polyps or masses identified during a colonoscopy should be marked within 2 weeks of surgical intervention.

Pelvic MRI is the most accurate diagnostic tool, providing accurate loco-regional staging and effectively measuring the distance of cancer from the anorectal junction or anal verge [9]. It also effectively analyses soft tissues, including the mesorectal fascia, circumferential resection margins, tumor penetration depth, and local lymphadenopathy [10]. Endorectal ultrasound is equally sensitive for early clinical staging of rectal tumors and the detection of potential sphincter infiltration [11]. A thoraco-abdominal CT scan is useful for identifying distant metastases. Diagnosis should include a serum measurement of carcinoembriyogenic antigen (CEA), mismatch repair or microsatellite instability testing, and evaluation of genetic mutations such as RAS and BRAF, particularly in the context of potential Lynch syndrome [12]. A PET-CT scan may rule out distant metastases in patients with extensive extramural invasive disease indicated on MRI, high levels of CEA at presentation, or suspected liver metastases on a CT scan. However, PET-CT should not be considered an essential diagnostic tool [13].

Ideally, every rectal cancer patient should undergo a complete colonoscopy, a pelvic MRI, and a thoraco-abdominal CT scan, in order to obtain a proper tumor staging. Colonoscopy is the first step in diagnosing rectal cancer, and is essential in order to rule out synchronous lesions. When an obstructing rectal cancer prevents the endoscope from passing through, a colon CT scan, a virtual colon CT scan, or virtual colonoscopy can be proposed to the patient. Pelvic MRI is the gold standard for the local staging of rectal cancer, whereas a CT scan is useful to rule out distant metastasis. Endorectal ultrasound is an adjunct tool to define the rectal wall extension of the cancer, especially in the early stages. The combination of all these diagnostic modalities is extremely useful for the clinician, but at the same time, can be extremely demanding for a frail older patient. For instance, complete colonoscopy requires a bowel preparation with the risk of dehydration. Pelvic MRI, as well as virtual colonoscopy, require some degree of patient cooperation in order to obtain a proper quality of the images. Therefore, the tolerance of the frail patient should always be kept in mind by the multidisciplinary team evaluating the case and deciding the best approach.

Age-specific diagnostic criteria and outcomes are summarized in Table 1.

## 3. Management: The Challenges of Advanced Age

In dealing with rectal cancer in the elderly population, treatment must also consider the patient’s life expectancy and therapeutic tolerance [14]. However, treatment options for geriatric patients in good health are not significantly different from those for the general population [15]. Talking generally about older adults means nothing and everything, since we refer to a very heterogeneous population, varying from fit patients to extremely frail individuals, and we cannot rely only on chronological age when making complex decisions for patients.

The ideal management of rectal cancer in fit patients derives from clinical trials and depends on the disease stage. However, frail older adults have not been included in these trials, so the ideal management in this group of patients is still a matter of debate, and should be tailored as much as possible to every single individual. Trials in older patients are challenging to complete, as the Alliance N0949 trial demonstrated, by failing to be completed due to poor accrual [16]. Unfortunately, several studies demonstrate significant disparities in therapeutic interventions concerning elderly patients, with a marked decline in the likelihood of these individuals receiving guideline-recommended treatments as age increases [17]. This phenomenon can be attributed to the current insufficient representation of this demographic in clinical trials; the absence of consistent geriatric assessments even in scheduled medical contexts, which become virtually non-existent in urgent care settings; and a general lack of knowledge regarding appropriate and effective geriatric care. Furthermore, an ethical concern arises from the not-uncommon practice of making clinical–surgical decisions in alignment with the preferences and beliefs of the patient’s family regarding the perceived value of aggressive treatment, rather than engaging in direct consultation with the patients themselves [18].

Regardless of the disease stage, international guidelines recommend, for every older adult diagnosed with rectal cancer, a geriatric assessment before deciding on the ideal treatment, as suggested by the International Society of Geriatric Oncology [19] and the American Society of Clinical Oncology [20]. The most frequently reported tailored assessments in the geriatric rectal cancer setting include the Comprehensive Geriatric Assessment, frailty assessment, the Cancer and Aging Research Group (CARG) score, the Chemotherapy Risk Assessment Scale for High age (CRASH) score, and the Age-adjusted Charlson Comorbidity Index (ACCI). The major scoring systems are summarized in Table 2.

Ultimately, a comprehensive evaluation by a multidisciplinary team consisting of radiologists, surgeons, oncologists, radiation oncologists, and pathologists is paramount in order to decide the optimal therapeutic approach and assess each patient’s operative risk [21]. A multidisciplinary evaluation is crucial for personalized treatment and better patient outcomes [22,23].

Furthermore, elderly patients should undergo a complete geriatric assessment to evaluate frailty, vulnerability, and the potential futility of a surgical approach [24]. Age itself should not be considered a determining factor for excluding specific therapeutic options; instead, it should be considered alongside the patient’s comorbidities, functional status, physiological decrease in homeostatic responses, and social–cultural–economic factors [25]. A proper assessment prevents or mitigates postoperative morbidities and delirium, allows better planning of the proper postoperative setting for the patient, and promotes faster recovery, even in selected geriatric patients, through the enhanced recovery after surgery (ERAS) protocol [26], which emphasizes early mobilization, oral feeding, and the use of non-opioid analgesics [27].

**Figure 1:**
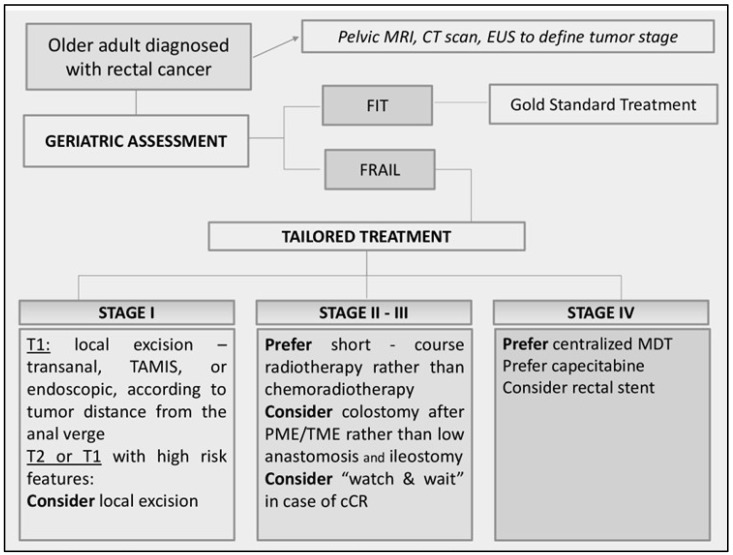
Flowchart of management of older adults diagnosed with rectal cancer. MRI: Magnetic resonance imaging. CT Scan: computed tomography scan; EUS: endoscopic ultrasound; TAMIS: transanal minimally invasive surgery; PME/TME: partial/total mesorectal excision; cCR complete clinical response; MDT: multi disciplinary team.

Therapeutic strategies in frail older adults vary according to cancer stage [28]. A flowchart of the possible management strategies can be summarized as follows (Figure 1).

- Stage I

T1 N0, no high-risk features. It is already established that in selected patients, total mesorectal excision (TME) can be avoided in favor of a less invasive approach, namely for T1 N0 lesions extending for less than 40–50% of the rectal circumference, with a diameter of 3 cm or less, not extending beyond the upper third of the submucosal layer, exhibiting well-to moderately differentiated morphology, and without signs of neurovascular invasion. Morino et al. demonstrated a significantly higher local recurrence rate for Sm2-3 tumors [29], while Doornebosch et al. found local recurrence rates at 3 years for tumors 3 cm in size or smaller to be significantly lower than for tumors larger than 3 cm [30]. Finally, Borschitz et al. identified histopathological features as risk factors for local recurrence [31]. Therefore, a surgical transanal excision or TAMIS (transanal minimally invasive surgery) excision, or an endoscopic polypectomy, depending on the tumor distance from the anal verge and polyp characteristics, can be deemed satisfactory in this particular subset of patients. If histological findings align with the initial clinical diagnosis, the patient can follow a surveillance program [32].

T1-2 N0. T1 tumors exhibiting high-risk features or T2 lesions have a local recurrence rate of 11–45% following simple transanal excision, making transabdominal rectal resection with partial or total mesorectal excision the preferred treatment.

In high-risk patients, local excision with the previously reported techniques is an alternative to transabdominal resection, to be considered exclusively for patients who refuse invasive surgical approaches or are deemed unfit for surgery, fragile, or at high-risk for complications at the geriatric assessment [33]. This decision should be taken by a tumor board with the involvement of a geriatrician.

In a multicenter, phase II trial, Stijns et al. explored the long-term outcomes of chemoradiotherapy followed by organ-sparing local excision in patients with early-stage rectal cancer, demonstrating that almost two-thirds of patients could achieve organ preservation, along with good long-term oncological outcomes [34]. In a randomized trial enrolling 55 patients with rectal cancer at stage T2 or lower, Bach et al. compared radical surgery versus transanal endoscopic microsurgery. Organ preservation was obtained in 70% of patients, with no difference in overall and disease-free survival at 4 years between groups. The authors concluded that the presented data support the use of organ preservation for patients considered unsuitable for primary total mesorectal excision [35].

- Stage II and III

Neoadjuvant therapy is the gold-standard treatment. The preferred scheme requires the patient to undergo neoadjuvant chemoradiotherapy, followed by TME at 8 to 12 weeks. This ideal approach has been demonstrated to reduce the local recurrence rate and to improve sphincter preservation. Alternative neoadjuvant treatments include short-course radiotherapy and total neoadjuvant treatment.

Geriatric patients, with their lower physiological ability to adequately respond to stressful events, tend to be more fragile and prone to complications, perioperative death, and loss of autonomy in activities of daily living (ADLs), and are more inclined to suffer from the toxic effects of radiochemotherapy [36]. For elderly patients with stage II and III resectable rectal cancer, both preoperative neoadjuvant short-course RT and standard neoadjuvant CRT demonstrate effectiveness, yielding comparable rates of overall survival, disease-free survival, local recurrence, sphincter preservation, and distant metastases [37]. However, short-course RT is associated with a relevant lower toxicity, and when combined with a delayed surgical program (at least 4 weeks post-RT), it is regarded as the preferred treatment option for frail patients [38]. In these patients, the primary objective is not survival per se, but rather the preservation of pre-treatment performance status and quality of life [39].

Some patterns of oncologic medical treatment have not been extensively tested on geriatric patients over 70 years of age, given the generally higher toxicity associated with radiation and chemotherapy in this demographic [40]. Only one-third of elderly patients receive both surgical intervention and CRT based on tumor stage; more than 50% undergo solely surgical treatment, while approximately 15% are provided with palliative care only [41]. The disparity in treatment approaches is particularly pronounced when comparing patients under and over 70 years of age: while more than half of patients under 70 receive both surgical intervention and CRT, less than 10% of those over 70 have access to this therapeutic combination, which 73% of older patients receive exclusively surgical treatment, in contrast to their younger counterparts [42].

Both surgical and medical therapy can result in long-term adverse consequences, such as chronic pain, fecal incontinence, and sexual dysfunction [43].

Furthermore, frail and elderly patients are often subject to undertreatment, palliative care, or overtreatment, which may result in overall higher mortality rates, reduced survival outcomes, and impaired functional status. Vulnerable, yet not frail, elderly patients may benefit from personalized treatment approaches, such as reduced initial doses of CRT or less-invasive surgical techniques [44]. Conversely, fit patients may benefit from standard treatment protocols [45].

Some patients achieve a complete clinical response to neoadjuvant therapy, with no evidence of residual disease on clinical examination, MRI, or endoscopy/anoscopy with biopsies. In such cases, a “watch-and-wait” approach, without surgical intervention but only with strict surveillance, has been described [46]. In the ideal fit patient, the watch-and-wait strategy can only be proposed in the context of clinical trials performed in experienced centers or if the patient refuses the TME surgery. However, in a frail older adult attaining a complete clinical response, the risk of postoperative complications should be taken into account. It is not usual, in this subgroup of patients, especially for those with severe comorbidities, for the surgeon to decide to avoid a complex and high-risk low colorectal or colo-anal anastomosis, and thus perform a definitive colostomy. All these surgical aspects should be balanced with the risk of recurrence that accompanies the watch-and-wait strategy. In 80-year-old patients, the 1-year survival rate has been reported to be better after the watch-and-wait strategy as compared to after TME surgery [47]. Moreover, salvage surgery after watch-and-wait has been reported to be possible in more than 90% of recurrences, leading to local disease control in 94% and organ preservation in 78%, and this is an additional aspect to take into account in the decision-making for a frail patient who has obtained a complete clinical response [48].

- Stage IV

Given their exclusion from surveillance screening protocols, older adults are more prone to being diagnosed with metastatic rectal cancer and a large primary tumor, causing symptoms such as bowel obstruction or bleeding. In a meta-analysis, stenting demonstrated a lower effectiveness in relieving the obstruction as compared with palliative surgery, but a lower 30-day mortality [49]. Therefore, stenting is a reasonable option in frail older adults. The standard chemotherapy regimen for unresectable stage IV rectal cancer is FOLFOX or FOLFIRI, with the aim of prolonging overall survival and maintaining quality of life. An EGFR inhibitor is added if the tumor is wild-type for KRAS, NRAS, and BRAF. Moreover, the VEGF-inhibiting antibody bevacizumab has demonstrated a survival benefit in older adults when added to a fluoropyrimidine, as compared to irinotecan or oxaliplatin [50]. Capecitabine as an oral agent does not require intravenous access and frequent clinic visits, and this should be taken into account in frail older adults with no or limited support from relatives or caregivers. The CAIRO 3 study showed that if the induction treatment succeeded, maintenance treatment with capecitabine and bevacizumab had a lower progression-free survival rate than observation alone. However, in the over 70-year old patient group, this benefit was not statistically significant [51]. Finally, sporadic microsatellite instability tumors occur in older adults, and results with immunotherapies are promising in frail patients due to their low toxicity profile [52,53].

Patients with potentially resectable metastatic rectal cancer should be accurately evaluated with a centralized multidisciplinary team assessment. The real-life RAXO study demonstrated that older adults with metastatic colorectal cancer had a 25% rate of upfront resectability at a centralized MDT assessment, as compared to the 12% at a localized MDT assessment. Interestingly, those older adults undergoing curative-intent surgery reported a median overall survival of 67 months [54].

## 4. Surgery in Frail Patients: The Great Challenge of the Modern Era

The operative management of rectal cancer depends on the type, location, and extent of the tumor [55]. In the ideal patient, rectal resection with total or partial mesorectal excision or abdominoperineal resection (APR) are the gold-standard treatments, depending on tumor size, distance from the anal verge, and preoperative chemoradiation.

Surgery for rectal cancer in elderly patients, and especially in frail elderly patients, is burdened by well-known increased perioperative comorbidities and mortality rates. Age alone is associated with increased mortality following elective colorectal resection, by up to 15.6% in patients >80 years of age [56]. Faiz et al. demonstrated a more than doubled 30-day mortality risk in patients aged 85 years or older than in patients ten years younger (between 75 and 84 years), with an OR of 2.47 (*p* < 0.001) [57]. Older individuals are also more prone to an unfavorable postoperative course. Cardiovascular complications increase significantly with age, while pulmonary complications are twice as common in older vs. younger patients [58].

The preoperative assessment of these patients cannot be exempted from a thorough frailty screening. The available literature is consistent in identifying comorbidities as an independent factor of postoperative complications and mortality. A retrospective analysis of 29,733 patients older than 67 years with stage 1–3 colorectal cancer demonstrated increased mortality associated with heart failure, chronic obstructive pulmonary disease, and diabetes mellitus. Also, sarcopenia, which is present in 11 to 50% of patients aged 80 years or older, is associated with decreased survival and higher postoperative complication rates. Lieffers et al. found sarcopenia to be an independent predictor of postoperative length of stay, infection, and rehabilitation care [59,60]. Frail individuals tend to experience higher rates of postoperative complications (up to 65%), prolonged hospital stays, increased readmission rates, and, consequently, elevated healthcare costs [61]. As such, surgeons should utilize preoperative patient stratification and surgical risk scores to inform their decision-making [62]. Several validated scores are available, including the P-Possum score, the ACS-NSQUIP score, and the Multidimensional Frailty Score (MFS), which can help to predict postoperative complications and mortality [63].

Indeed, older patients with rectal cancer are less likely to undergo surgery as compared to their younger counterparts. Moreover, reports show they undergo less aggressive operations, leading to lower R0 resections and lower rates of specimens with at least 12 lymph nodes retrieved at the final pathology examination [2].

Another critical issue to consider when dealing with this subset of patients is the increased stoma rate. As previously mentioned, frail older adults have an increased rate of postoperative complications, including an increased rate of anastomotic leaks due to their reduced physiological reserve. This aspect, together with the tendency to face lower and larger cancers in the elderly, pushes surgeons to avoid a colorectal anastomosis or to protect it with a diverting stoma. Issues related to stomas in the elderly are significant, and include fluid loss, dehydration, prolonged hospitalization, loss of autonomy, and negative psychological impacts [64]. With this perspective, a colostomy, even if definitive, seems preferable to an ileostomy.

Minimally invasive approaches for colorectal resections in the elderly have been strongly debated, and sometimes ostracized. The major concern regarding the adoption of minimally invasive techniques is the application of prolonged pneumoperitoneum, prolonged Trendelemburg, and prolonged operative time in elderly patients with a reduced physiological reserve. With growing data and experience, the laparoscopic approach has been demonstrated to be feasible, suggesting that minimally invasive rectal resection should be employed whenever possible [65]. Laparoscopy allows for faster restoration of bowel function, shorter hospital stays, improved cosmetic outcomes, and similar rates of surgical radicality, postoperative morbidity, mortality, and recurrence rates [66]. Interestingly, the benefits of laparoscopic rectal resection in terms of postoperative mortality and postoperative major complications have been reported to be greater for older adults than for younger adults [67]. Similar conclusions can be applied to robot-assisted procedures when compared to laparoscopy, suggesting an interesting role for the robotic approach, which also guarantees to reduce the extreme Trendelemburg position and to reduce the pneumoperitoneum [68]. Major studies investigating a minimally invasive approach to rectal cancer in older adults are reported in Table 3.

## 5. Conclusions

The management of rectal cancer presents unique challenges, particularly in the context of the aging population, where factors such as physiological resilience and pre-existing conditions necessitate careful consideration. Effective treatment regimens include surgical options ranging from minimally invasive procedures to more extensive resections, depending on tumor characteristics and staging. The implementation of multidisciplinary teams is critical for optimizing therapeutic strategies and promoting patient-centric care. A lot has to be achieved in this setting. Several studies report that the majority of cancer-related deaths in older patients affected by rectal cancer are in the first postoperative year. The aim of the present review is to better define the current evidence in this particular setting, and to provide some patient-tailored strategies according to the rectal cancer stage and the geriatric assessment of the patient. If the patient is deemed to be frail, local excision should be the treatment of choice in the early stages, and should also be considered “off-label” in the case of T2 tumors. In stage II or III frail rectal cancer patients, short-course radiotherapy should be the first-line approach, according to current evidence. However, if the patient is submitted to standard long-course treatment and obtains a complete clinical response, the watch-and-wait strategy should be seriously considered. Concerning rectal cancer surgery, low anastomosis and ileostomies are at high risk of complications in this subset of patients, and a definitive colostomy after rectal resection could be an acceptable compromise. Overall, ongoing research is vital in order to enhance our understanding of rectal cancer and develop targeted therapies that can better address individual patient needs, particularly as the demographic landscape continues to shift. Future studies should aim to explore molecular markers and refine personalized treatment approaches in the geriatric, oncologic, endoscopic, and surgical settings, paving the way for improved management of rectal cancer.

## Figures and Tables

**Table 1:** Age-specific diagnostic criteria and outcomes in rectal cancer. EUS: endoscopic ultrasound; MRI: magnetic resonance imaging; CT scan: computed tomography scan.

Age Group	Screening	Common Cancer Characteristics	Diagnosis	Outcomes Reported [2]
65 years or lower	Screening protocol starting at 50. One-time colonoscopy recommended, or fecal occult blood test every 2 years	Typically early-stage disease, often asymptomatic	All diagnostic tools should be employed: colonoscopy, EUS, pelvic MRI, thoraco-abdominal CT scan	No surgery—10% Neoadjuvant RT—50% R0 resection—94% Adjuvant chemotherapy—50%
65–79 years	Fecal occult blood test every 2 years; colonoscopy if symptomatic	Tumors often present with rectal bleeding or changes in bowel habits	Ideally, all diagnostic tools should be employed, but diagnostic complexity increases with comorbidities and frailty	No surgery—16% Neoadjuvant RT—38% R0 resection—95% Adjuvant chemotherapy—38%
≥80 years	Screening not routinely recommended; diagnostic workup based on symptoms	Tumors tend to be located closer to anal verge	Increased frailty, diminished family support, and advanced age complicate diagnosis. Evaluate patient’s tolerance to diagnostic tools	No surgery—29% Neoadjuvant RT—23% R0 Resection—88% Adjuvant chemotherapy—7%

**Table 2:** Characteristics of major frailty scoring systems in elderly cancer patients.

Scoring Systems	Advantages	Disadvantages
Comprehensive Geriatric Assessment (CGA)	Assesses physical and cognitive vulnerabilities.	Complex to use in emergency setting; Time-consuming to administer; Necessitates a multidisciplinary team.
Clinical Frailty Scale (CFS)	Evaluates patient autonomy; Quick to administer; Applicable for screening purposes.	Non-standardizable (inter-observer variation).
Cancer and Aging Research Group (CARG)	Quick to administer; Evaluates social aspect of life; Assesses chemotherapy toxicity.	Specifically designed for oncological patients.
Chemotherapy Risk Assessment Scale for High Age (CRASH)	Provides detailed predictions on different types of toxicity (hematologic, non-hematologic, combined); Includes cognitive, nutritional, and social assessment.	Specifically designed for oncological patients; Time-consuming to administer.
Age-adjusted Charlson Comorbidity Index (ACCI)	Long-term mortality predictor; Simple to calculate.	Does not assess patient’s social and cognitive factors; Does not directly assess frailty.

**Table 3:** Major studies investigating a minimally invasive approach to rectal cancer in older adults.

Authors	Year	Study Design	Study Population	Major Findings
Chesney, et al. [67]	2020	Single-center cohort study. Patients operated on due to sigmoid and rectal cancer between 2002 and 2018. Assessment of 30-day postoperative outcomes comparing age group and surgical approach.	293 older adults (70 years or older) and 499 younger adults. Laparoscopy rate: 41%.	Minimally invasive approach was associated with greater reduction in deaths and major complications (Clavien–Dindo III_IV) in older adults than in younger adults.
Zhang, et al. [66]	2021	Single-center study of consecutive patients aged 80 years or older, operated on due to rectal cancer between 2007 and 2015. Outcome: safety and effectiveness of laparoscopy.	45 laparoscopic rectal resections and 39 open rectal resections were compared.	Laparoscopic rectal resection was significantly associated with the following: longer operatory time, less intraoperative blood loss, shorter time to diet recovery and hospital stay, lower postoperative complications and wound infection rate.
Ali M, et al. [68]	2023	Single-center study cohort of patients operated on with minimally invasive (robotic/laparoscopic) rectal resection between 2019 and 2022. Outcome: postoperative complications according to age group and surgical approach.	108 older adults: 45 robotic rectal resections and 63 laparoscopic. 190 younger adults: 85 robotic rectal resections and 105 laparoscopic.	Younger patients experience lower Clavien–Dindo grades of complications; Clavien–Dindo III-IV are seen more frequently in laparoscopic rectal surgery than in robotics.

## References

[1] Howlander N., Noone A., Krapcho M., Miller D., Brest A.Y., Ruhl J., Tatalovich Z., Mariotto A., Lewis D. (2016). Cancer Statistics Review 1975–2014—SEER Statistics, National Cancer Institute. SEER Cancer Statistics Review 1975–2014.

[2] Mourad A.P., De Robles M.S., Putnis S., Winn R.D. (2021). Current treatment approaches and outcomes in the management of rectal cancer above the age of 80. Curr. Oncol..

[3] Steele R.J., Rey J.F., Lambert R. (2012). European guidelines for quality assurance in colorectal cancer screening and diagnosis. First Edition-Professional requirements and training. Endoscopy.

[4] Bretthauer M., Kaminski M.F., Hassan C., Kalagher M., Holme O., Hoff G., Loberg M., Regula J., Castells A., Adami H.O. (2017). America, we are confused: The updated US PSTF recommendation on colorectal cancer screening. Ann. Int. Med..

[5] van Hees F., Habbema J.D., Meester R.G., Lansdorp-Vogelaar I., van Ballegooijen M., Zauber A.G. (2014). Should colorectal cancer screening be considered in elderly persons without previous screening? A cost-effectiveness analysis. Ann. Int. Med..

[6] Kamiński M.F., Hassan C., Bisschops R., Pohl J., Pellisé M., Dekker E., Ignjatovic-Wilson A., Hoffmann A., Longcroft-Wheaton G., Heresbach D. (2014). Advanced imaging for detection and differentiation of colorectal neoplasia: European Society of Gastrointestinal Endoscopy (ESGE) Guideline. Endoscopy.

[7] Hazewinkel Y., Lopez-Ceron M., East J.E., Rastogi A., Pellisé M., Nakajima T., van Eeden S., Tytgat K., Fockens P., Dekker E. (2013). Endoscopic features of sessile serrated adenomas: Validation by international experts using high-resolution white-light endoscopy and narrow-band imaging. Gastrointest. Endosc..

[8] D’Souza N., tot Babberich M.P.D.N., d’ Hoore A., Tiret E., Xynos E., Beets-Tan R.G.H., Nagtegaal I.D., Blomqvist L., Holm T., Glimelius B. (2019). Definition of the Rectum: An International, Expert-based Delphi Consensus. Ann. Surg..

[9] Beets-Tan R.G.H., Lambregts D.M.J., Maas M., Bipat S., Barbaro B., Curvo-Semedo L., Fenlon H.M., Gollub M.J., Gourtsoyianni S., Halligan S. (2018). Magnetic resonance imaging for clinical management of rectal cancer: Updated recommendations from the 2016 European Society of Gastrointestinal and Abdominal Radiology (ESGAR) consensus meeting. Eur. Radiol..

[10] Al-Sukhni E., Milot L., Fruitman M., Beyene J., Victor J.C., Schmocker S., Brown G., McLeod R., Kennedy E. (2012). Diagnostic accuracy of MRI for assessment of T category, lymph node metastases, and circumferential resection margin involvement in patients with rectal cancer: A systematic review and meta-analysis. Ann. Surg. Oncol..

[11] Burdan F., Sudol-Szopinska I., Staroslawska E., Kolodziejczak M., Klepacz R., Mocarska A., Caban M., Zelazowska-Cieslinska I., Szumilo J. (2015). Magnetic resonance imaging and endorectal ultrasound for diagnosis of rectal lesions. Eur. J. Med. Res..

[12] Guinney J., Dienstmann R., Wang X., de Reynies A., Schlicker A., Soneson C., Marisa L., Roepman P., Nyamundanda G., Angelino P. (2015). The consensus molecular subtypes of colorectal cancer. Nat. Med..

[13] Van Cutsem E., Cervantes A., Adam R., Sobrero A., Van Krieken K., Aderka D., Aranda Aguilar E., Bardelli A., Benson A., Bodoky G. (2016). ESMO consensus guidelines for the management of patients with metastatic colorectal cancer. Ann. Oncol..

[14] Huang C.K., Shih C.H., Kao Y.S. (2024). Elderly Rectal Cancer: An Updated Review. Curr. Oncol. Rep..

[15] Glynne-Jones R., Wyrwicz L., Tiret E., Brown G., Rödel C., Cervantes A., Arnold D., ESMO Guidelines Committee (2017). Rectal cancer: ESMO Clinical Practice Guidelines for diagnosis, treatment and follow-up. Ann. Oncol..

[16] McCleary N.J., Hubbard J., Mahoney M.R., Meyerhardt J.A., Sargent D., Venook A., Grothey A. (2018). Challenges of conducting a prospective clinical trial for older patients: Lessons learned from NCCTG N0949 (alliance). J. Geriatr. Oncol..

[17] Adelson P., Fusco K., Karapetis C., Wattchow D., Joshi R., Price T., Sharplin G., Roder D. (2018). Use of guideline-recommended adjuvant therapies and survival outcomes for people with colorectal cancer at tertiary referral hospitals in South Australia. J. Eval. Clin. Pract..

[18] Chen R.C., Royce T.J., Extermann M., Reeve B.B. (2012). Impact of age and comorbidity on treatment and outcomes in elderly cancer patients. Semin. Radiat. Oncol..

[19] Montroni I., Uglini G., Saur N.M., Spinelli A., Rostoft S., Millan M., Wolthuis A., Daniels I.R., Hompes R., Penna M. (2018). Personalized management of rectal cancer: Expert recommendations from the European Society of Surgical Oncology, European Society of Coloproctology, International Society of Geriatric Oncology, and American College of Surgeons Commission on Cancer. Eur. J. Surg. Oncol..

[20] Mohile S.G., Dale W., Somerfield M.R., Schonberg M.A., Boyd C.M., Burhenn P.S., Canin B., Cohen H.J., Holmes H.M., Hopkins J.O. (2018). Practical Assessment and Management of Vulnerabilities in Older Patients Receiving Chemotherapy: ASCO Guideline for Geriatric Oncology. J. Clin. Oncol..

[21] Munro A., Brown M., Niblock P., Steele R., Carey F. (2015). Do multidisciplinary team (MDT) processes influence survival in patients with colorectal cancer? A population-based experience. BMC Cancer.

[22] Richardson B., Preskitt J., Lichliter W., Peschka S., Carmack S., de Prisco G., Fleshman J. (2016). The effect of multidisciplinary teams for rectal cancer on delivery of care and patient outcome: Has the use of multidisciplinary teams for rectal cancer affected the utilization of available resources, proportion of patients meeting the standard of care, and does this translate into changes in patient outcome?. Am. J. Surg..

[23] Podda M., Sylla P., Baiocchi G., Adamina M., Agnoletti V., Agresta F., Ansaloni L., Arezzo A., Avenia N., Biffl W. (2021). Multidisciplinary management of elderly patients with rectal cancer: Recommendations from the SICG, SIFIPAC, SICE, and the WSES International Consensus Project. World J. Emerg. Surg..

[24] Mason M.C., Crees A.l., Dean M.R., Bashir N. (2018). Establishing a proactive geriatrician led comprehensive geriatric assessment in older emergency surgery patients: Outcomes of a pilot study. Int. J. Clin. Pract..

[25] Papamichael D., Audisio R.A., Glimelius B., de Gramont A., Glynne-Jones R., Haller D., Kohne C.-H., Rostoft S., Lemmens V., Mitry E. (2015). Treatment of colorectal cancer in older patients: International Society of Geriatric Oncology (SIOG) consensus recommendations 2013. Ann. Oncol..

[26] Pedziwiatr M., Pisarska M., Major P., Grochowska A., Matłok M., Przęczek K., Stefura T., Budzyński A., Kłęk S. (2016). Laparoscopic colorectal cancer surgery combined with enhanced recovery after surgery protocol (ERAS) reduces the negative impact of sarcopenia on short-term outcomes. Eur. J. Surg. Oncol..

[27] Duron J.J., Duron E., Dugue T., Grochowska A., Matlok M., Przeczek K., Stefura T., Budzinxki A., Klek S. (2011). Risk factors for mortality in major digestive surgery in the elderly: A multicenter prospective study. Ann. Surg..

[28] Sheikh A.R., Yameen H., Hartshorn K. (2018). Treatment of Rectal Cancer in Older Adults. Curr. Oncol. Rep..

[29] Morino M., Allaix M.E., Caldart M., Scozzari G., Arezzo A. (2011). Risk factors for recurrence after transanal endoscopic microsurgery for rectal malignant neoplasm. Surg. Endosc..

[30] Doornebosch P.G., Zeestraten E., de Graaf E.J., Hermsen P., Dawson I., Tollenaar R.A., Morreau H. (2012). Transanal endoscopic microsurgery for T1 rectal cancer: Size matters!. Surg. Endosc..

[31] Borschitz T., Heintz A., Junginger T. (2006). The influence of histopathologic criteria on the long-term prognosis of locally excised pT1 rectal carcinomas: Results of local excision (transanal endoscopic microsurgery) and immediate reoperation. Dis. Colon Rectum.

[32] Veereman G., Joan Vlayen J., Jo Robays J., Fairon N., Stordeur S., Rolfo C., Bielen D., Bols A., Demetter P., D’hoore A. (2017). Systematic review and meta-analysis of local resection or transanal endoscopic microsurgery versus radical resection in stage I rectal cancer: A real standard?. Crit. Rev. Oncol. Hematol..

[33] Garcia-Aguilar J., Renfro L.A., Chow O.S., Shi Q., Carrero X.Q., Lynn P.B., Thomas C., Chan E., Cataldo P.A., Marcet J.E. (2015). Organ preservation for clinical T2N0 distal rectal cancer using neoadjuvant chemoradiotherapy and local excision (ACOSOG Z6041): Results of an open-label, single-arm, multi-institutional, phase 2 trial. Lancet Oncol..

[34] Stijns R.C.H., de Graaf E.J.R., Punt C.J.A., Nagtegaal I.D., Nuyttens J.J.M.E., van Meerten E., Tanis P.J., de Hingh I.H.J.T., van der Schelling G.P., Acherman Y. (2019). Long-term Oncological and Functional Outcomes of Chemoradiotherapy Followed by Organ-Sparing Transanal Endoscopic Microsurgery for Distal Rectal Cancer: The CARTS Study. JAMA Surg..

[35] Bach S.P., Gilbert A., Brock K., Korsgen S., Geh I., Hill J., Gill T., Hainsworth P., Tutton M.G., Khan J. (2021). Radical surgery versus organ preservation via short-course radiotherapy followed by transanal endoscopic microsurgery for early-stage rectal cancer (TREC): A randomised, open-label feasibility study. Lancet Gastroenterol. Hepatol..

[36] Poillucci G., Ortenzi M., Pilia T., Murzi V., Di Saverio S., Segalini E., Locci E., Cois A., Pisanu A., Podda M. (2023). Updates on the multidisciplinary management of elderly patients with rectal cancer: A narrative review. Minerva Surg..

[37] Qiaoli W., Yongping H., Wei X., Guoqiang X., Yunhe J., Qiuyan L., Cheng L., Mengling G., Jiayi L., Wei X. (2019). Preoperative short-course radiotherapy (5 × 5 Gy) with delayed surgery versus preoperative long-course radiotherapy for locally resectable rectal cancer: A meta-analysis. Int. J. Color. Dis..

[38] Francois E., Pernot M., Ronchin P., Nouhaud E., Lafay I.M., Pascal A., Clavere P., Vendrely V., Boige V., Thamphya B. (2021). NACRE: A randomized study comparing short course radiotherapy with radiochemotherapy for locally advanced rectal cancers in the elderly—Preliminary results. J. Clin. Oncol..

[39] Stegmann M.E., Festen S., Brandenbarg D., Schuling J., van Leeuwen B., de Graeff P., Berendsen A.J. (2019). Using the Outcome Prioritization Tool (OPT) to assess the preferences of older patients in clinical decision making: A review. Maturitas.

[40] De Felice F., Crocetti D., Maiuri V., Parisi M., Marampom F., Izzo L., de Toma G., Musio D., Tombolini V. (2020). Locally Advanced Rectal Cancer: Treatment Approach in Elderly Patients. Curr. Treat. Options Oncol..

[41] Koroukian S.M., Xu F., Bakaki P.M., Diaz-Insua M., Towe T.P., Owusu C. (2010). Comorbidities, functional limitations, and geriatric syndromes in relation to treatment and survival patterns among elders with colorectal cancer. J. Gerontol. A Biol. Sci. Med. Sci..

[42] Janssen-Heijnen M.L., Houterman S., Lemmens V.E., Louwman M.W.J., Maas H.A.A.M., Coebergh J.W.W. (2005). Prognostic impact of increasing age and co-morbidity in cancer patients: A population-based approach. Crit. Rev. Oncol. Hematol..

[43] Downing A., Morris E.J., Richards M., Corner J., Wright P., Sebag-Montefiore D., Finan P., Kind P., Wood C., Lawton S. (2015). Health-related quality of life after colorectal cancer in England: A patient-reported outcomes study of individuals 12 to 36 months after diagnosis. J. Clin. Oncol..

[44] Hathout L., Maloney-Patel N., Malhotra U., Wang S.J., Chokhavatia S., Dalal I., Poplin E., Jabbour S.K. (2018). Management of locally advanced rectal cancer in the elderly: A critical review and algorithm. J. Gastrointest. Oncol..

[45] Buntinx F., Campbell C., van den Akker M. (2014). Cancer in the elderly. J. Cancer Epidemiol..

[46] Glynne-Jones R., Hughes R. (2012). Critical appraisal of the ‘wait and see’ approach in rectal cancer for clinical complete responders after chemoradiation. Br. J. Surg..

[47] Smith F.M., Rao C., Oliva Perez R., Bujko K., Athanasiou T., Habr-Gama A., Faiz O. (2015). Avoiding radical surgery improves early survival in elderly patients with rectal cancer, demonstrating complete clinical response after neoadjuvant therapy: Results of a decision-analytic model. Dis. Colon. Rectum..

[48] Habr-Gama A., Gama-Rodrigues J., São Julião G.P., Proscurshim I., Sabbagh C., Lynn P.B., Perez R.O. (2014). Local recurrence after complete clinical response and watch and wait in rectal cancer after neoadjuvant chemoradiation: Impact of salvage therapy on local disease control. Int. J. Radiat. Oncol. Biol. Phys..

[49] Zao X.D., Cai B.B., Cao R.S., Shi R.H. (2013). Palliative treatment for incurable malignant colorectal obstructions: A meta-analysis. World J. Gastroenterol..

[50] Landre T., Maillard E., Taleb C., Ghebriou D., Des Guetz G., Zelek L., Aparicio T. (2018). Impact of the addition of bevacizumab, oxaliplatin, or irinotecan to fluoropyrimidin in the first-line treatment of metastatic colorectal cancer in elderly patients. Int. J. Colorectal. Dis..

[51] Simkens L.H., van Tinteren H., May A., ten Tije A.J., Creemers G.J., Loosveld O.J.L., de Jongh F.E., Erdkamp F.L.G., Erjavec Z., van der Torren A.M.E. (2015). Maintenance treatment with capecitabine and bevacizumab in metastatic colorectal cancer (CAIRO3): A phase 3 randomised controlled trial of the Dutch Colorectal Cancer Group. Lancet.

[52] Franke A.J., Skelton W.P., Gaffar M., Lutfi F., Welniak S., Feely M., Starr J.S., Parekh H.D., Igbal A., Tan S. (2018). Differences in the characteristics of younger and older MSI-I colorectal cancer (CRC) as determined by universal reflex testing. J. Clin. Oncol..

[53] Elias R., Giobbie-Hurder A., McCleary N.J., Ott P., Hodi F.S., Rahma O. (2018). Efficacy of PD-1 & PD-L1 inhibitors in older adults: A meta-analysis. J. Immunother. Cancer.

[54] Lehtomäki K., Soveri L.M., Osterlund E., Lamminmäki A., Uutela A., Heervä E., Halonen P., Stedt H., Aho S., Muhonen T. (2023). Resectability, Resections, Survival Outcomes, and Quality of Life in Older Adult Patients with Metastatic Colorectal Cancer (the RAXO-Study). J. Clin. Med..

[55] Neuman H.B., O’Connor E.S., Weiss J., Loconte N.K., Greenblatt D.Y., Greenberg C.C., Smith M.A. (2013). Surgical treatment of colon cancer in patients aged 80 years and older: Analysis of 31,574 patients in the SEER-Medicare database. Cancer.

[56] Millan M., Merino S., Caro A., Feliu F., Escuder J., Francesch T. (2015). Treatment of colorectal cancer in the elderly. World J. Gastrointest. Oncol..

[57] Faiz O., Haji A., Bottle A., Clark S.K., Darzi A.W., Aylin P. (2011). Elective colonic surgery for cancer in the elderly: An investigation into postoperative mortality in English NHS hospitals between 1996 and 2007. Color. Dis..

[58] Scandrett K.G., Zuckerbraun B.S., Peitzman A.B. (2015). Operative risk stratification in the older adult. Surg. Clin. N. Am..

[59] Janssen I. (2011). The epidemiology of sarcopenia. Clin. Geriatr. Med..

[60] Lieffers J.R., Bathe O.F., Fassbender K., Winget M., Baracos V.E. (2012). Sarcopenia is associated with postoperative infection and delayed recovery from colorectal cancer resection surgery. Br. J. Cancer.

[61] Fagard K., Leonard S., Deschodt M., Devriendt E., Wolthuis A., Prenen H., Flamaing J., Milisen K., Wildiers H., Kenis C. (2016). The impact of frailty on postoperative outcomes in individuals aged 65 and over undergoing elective surgery for colorectal cancer: A systematic review. J. Geriatr. Oncol..

[62] Presley C.J., Krok-Schoen J.L., Wall S.A., Noonan A.M., Jones D.C., Folefac E., Williams N., Overcash J., Rosko A.E. (2020). Implementing a multidisciplinary approach for older adults with cancer: Geriatric oncology in practice. BMC Geriatr..

[63] Kim S.W., Han H.S., Jung H.W., Kim K.I., Hwang D.W., Kang S.B., Kim C.H. (2014). Multidimensional frailty score for the prediction of postoperative mortality risk. JAMA Surg..

[64] Yeo H.L., O’Mahoney P.R., Lachs M., Michelassi F., Mao J., Finlayson E., Abelson J.S., Sedrakyan A. (2016). Surgical Oncology outcomes in the aging US population. J. Surg. Res..

[65] Jiang J.B., Jiang K., Dai Y., Wang R.X., Wu W.Z., Wang J.J., Xie F.B., Li X.M. (2015). Laparoscopic versus open surgery for mid-low rectal cancer: A systematic review and meta-analysis on short-and long-term outcomes. J. Gastrointest. Surg..

[66] Zhang Q., Liang J., Chen J., Mei S., Wang Z. (2021). Outcomes of laparoscopic versus open surgery in elderly patients with rectal cancer. Asian Pac. J. Cancer Prev..

[67] Chesney T.R., Quereshy H.A., Draginov A., Chadi S.A., Quereshi F.A. (2020). Benefits of minimally invasive surgery for sigmoid and rectal cancer in older adults compared with younger adults: Do older adults have the most to gain?. J. Geriatr. Oncol..

[68] Ali M., Wang Y., Yu W., Baral S., Jun R., Wang D. (2023). Benefits of minimally invasive surgery for rectal cancer in older adults compared with younger adults: A retrospective study. J. Robot. Surg..

